# ZNF521 which is downregulated by miR-802 suppresses malignant progression of Hepatocellular Carcinoma through regulating Runx2 expression

**DOI:** 10.7150/jca.45190

**Published:** 2020-08-01

**Authors:** Nan Yang, Liang Wang, Tianxiang Chen, Runkun Liu, Zhikui Liu, Lei Zhang

**Affiliations:** 1 Department of Infectious Diseases, the First Affiliated Hospital of Xi'an Jiaotong University, Xi'an, Shaanxi, 710061, China.; 2 Department of Hepatobiliary Surgery, the First Affiliated Hospital of Xi'an Jiaotong University, No. 277 Yanta West Road, Xi'an 710061, China.; 3 Department of Geriatric Surgery, the First Affiliated Hospital of Xi'an Jiaotong University, Xi'an, Shaanxi, 710061, China.

**Keywords:** ZNF521, hepatocellular carcinoma, Runx2, miR-802, proliferation

## Abstract

Zinc finger protein 521 (ZNF521) plays an important role in the tumor development and process. However, its regulatory role in hepatocellular carcinoma (HCC) remains unclear. In this study, we demonstrated for the first time that ZNF521 mRNA and protein was down-regulated in HCC tissues and cell lines. Down-regulated ZNF521 expression was significantly associated with malignant prognostic features, including advanced TNM stage and large tumor size. For 5-year survival, ZNF521 served as a potential prognostic marker of HCC patients. Moreover, ZNF521 inhibited cell proliferation, colony formation and cell viability through Runx2 transcriptional inhibition and AKT phosphorylation pathway. Moreover, we demonstrated that ZNF521 expression was regulated by miR-802. In HCC tissues. MiR-802 has an inverse correlation with ZNF521 expression. In conclusion, we demonstrate for the first time that ZNF521 is down-regulated in HCC tissues and inhibits HCC growth through Runx2 transcriptional inhibition and AKT inactivation, which was regulated by miR-802, suggesting the potential therapeutic value for HCC.

## Introduction

Hepatocellular carcinoma (HCC) has been regarded as one of the leading causes of cancer-related death worldwide, especially in China 1. Although there have been considerable improvements in current therapy interventions for HCC, the clinical therapeutic outcomes are still undesirable because of its unlimited proliferation and tumor metastasis 2. Most of HCC patients are diagnosed at advanced stages due to lack of specific biomarkers and therapeutic targets, which leads to high mortality and poor prognosis 3. Therefore, investigating the molecular mechanisms involved in HCC pathogenesis is important for the treatment.

Zinc finger protein 521 (EHZF/ZNF521) is a multi-functional transcription co-factor containing 30 zinc fingers and an N-terminal motif that binds to the nucleosome remodeling and histone deacetylase (NuRD) complex. ZNF521 plays a critical role in the homeostasis of the hematopoietic progenitor cell compartment, neuronal differentiation, bone formation and cancer progression 4-6. ZNF521 can arrest the apoptosis and enhance the proliferation, migration, and invasion of gastric cancer cells via regulating miR-204-5p 7. ZNF521 interacts with GLI1 and GLI2 to enhance the activity of the sonic hedgehog pathway in medulloblastoma 8. Moreover, ZNF521 contributes to the clonogenic growth, migration and tumorigenicity of medulloblastoma cells 9. ZNF521 promotes B-cell viability and cyclin D1 gene expression in a B cell culture system 10. miR-9 promotes the neural differentiation of mouse bone marrow mesenchymal stem cells via targeting ZNF521 11. However, the potential role of ZNF521 in the pathogenesis of HCC has not been investigated.

In the present study, we found that ZNF521 was abnormally down-regulated both in HCC tissues and cells and that down-regulation was associated with malignant prognostic features and reduced survival of HCC patients. ZNF521 repressed the growth of HCC cells *in vitro* and *in vivo*. Furthermore, ZNF521 suppressed Rnux2 transcriptional activity and regulated Runx2-related PI3K/AKT signaling pathways. miR-802 regulated ZNF521 expression via binding to its 3'UTR in HCC cells. Therefore, our data confirm that ZNF521 could be as a potential strategy for the diagnosis and treatment of HCC.

## Materials and Methods

### Clinical specimens

Hepatocellular carcinomas (HCC) tissues and adjacent non-tumor tissues (>2 cm from the margin of tumor) were obtained from patients who received surgical resection at the First Affiliated Hospital of Xi'an Jiaotong University. All the specimens were snap-frozen in liquid nitrogen and kept at -80°C after surgical removal. This study was approved by the Ethics Committee of this hospital. The written informed consents had been obtained from all patients before study.

### Cell culture

All cell lines used in this study, including HCC cell lines (Hep3B, Huh7, SMMC-7721 and Bel-7402) and normal hepatic cell line (LO2) were obtained from the Institute of Biochemistry and Cell Biology (Chinese Academy of Sciences, Shanghai, China). Cell culture was performed in DMEM medium (Thermo Fisher, Grand Island, NY, USA) which containing 10% fetal bovine serum (FBS; Sigma-Aldrich, St. Louis, MO, USA) and 100 U/mL penicillin-streptomycin mixture (Beyotime Institute of Biotechnology, Haimen, China). The conditions for cell culture are as follows: a humidified atmosphere with 5% CO2 at 37°C.

### RNA extraction and quantitative real-time PCR (qRT-PCR)

The total RNA from HCC cells and tissues was extracted using TRIzol reagent (Invitrogen, Carlsbad, CA, USA) according to the manufacturer's protocol. cDNA was synthesized by TaqMan miRNA reverse transcription (Applied Biosystems, Foster City, CA, USA) and a PrimeScript Reverse Transcriptase kit (Takara, Dalian, China). The relative expression of miR-802 and ZNF521 mRNA were quantified using miRNA-specific TaqMan miRNA Assay Kit (Applied Biosystems) and the SYBR Premix Ex Taq™ Kit (Takara, Shiga, Japan) in the Applied Biosystems 7500 Sequence Detection system. Primers for miR-802 and ZNF521 were obtained from Genecopoeia (Guangzhou, China).

### Western blot

Immunohistochemical analysis status was performed as our previously publication 12. Cell lysates were prepared using RIPA lysis Buffer (RIPA; Pierce, Rockford, IL). Then protease inhibitors were added into lysates. A bicinchoninic acid (BCA) kit (Beyotime Institute of Biotechnology, Shanghai, China) was used to determine protein concentration. Subsequently, protein was separated in 10% sodium dodecyl sulfate polyacrylamide gel electrophoresis (SDS-PAGE) membrane and then transferred onto a polyvinylidene difluoride (PVDF) membrane (Mippore, Merck KGaA, Germany). Tris-buffered saline (TBS) containing 5% nonfat milk was used to block the membranes for an hour at room temperature. Thereafter, the membranes were incubated with specific primary antibodies at 4°C overnight. Subsequently, HRP-conjugated secondary antibody goat anti-rabbit IgG (1:2000, Abcam) was added and incubated for 2 h at room temperate. GAPDH was considered as the internal control. Bands signal were detected using the enhanced chemiluminescence (ECL; Millipore, Merck KGaA, Germany).

### Immunohistochemistry (IHC) staining and scoring

Immunohistochemical analysis status was performed as our previously publication 13, 14.

### Cell proliferation, cell cycle and viability detection

Cell Counting Kit-8 (CCK8) reagents (Dojindo, Kumamoto, Japan), EdU and colony formation were carried as described previously 14-16.

### Luciferase reporter assay

The sequence of ZNF521 3'-UTR containing the putative miR-802 binding region was amplified from human genomic DNA. Then the sequence was cloned into pGL3 luciferase reporter vector (Promega, Madison, WI, USA). The potential miR-802 binding sites were mutated by the Quick-change site-directed mutagenesis kit (Agilent Technologies, Santa Clara, CA, USA). The wild type (wt) ZNF521 3'-UTR vector or mutant (mt) ZNF521 3'-UTR vector and miR-802 mimics or miR-802 inhibitors were co-transfected into Hep3B cells by using Lipofectamine 2000 (Invitrogen, Carlsbad, CA, USA). The luciferase activity was measured using Dual-Luciferase Reporter Assay System (Promega, Madison, WI, USA) under luminometer (Berthold Detection System, Pforzheim, Germany), and luciferase activity was normalized to Renilla activity.

### *In vivo* experiments

Four-to-six-week-old male BALB/c nude mice (Centre of Laboratory Animals, The Medical College of Xi'an Jiaotong University, Xi'an, China) were used to establish the nude mouse xenograft model. Hep3B (5×10^6^) cells that were transfected with ZNF521 overexpression vectors or control vectors were mixed in 150 µL of Matrigel and were inoculated subcutaneously into the flank of nude mice. The tumor volume for each mouse was determined by measuring two of its dimensions and then calculated as tumor volume = length × width × width/2. After 3 weeks, the mice were sacrificed by cervical dislocation under anesthesia with ether and the xenograft tumor tissue was explanted for examination. Animal protocols were approved by the Institutional Animal Care and Use Committee of Xi'an Jiaotong University.

### Statistical analysis

In this study, statistical analyses were performed with SPSS 17.0 software (Abbott Laboratories, Chicago, IL). Data represent mean ± standard deviation (SD) of more than two independent experiments. A two-sided Student's t-test and one-way ANOVA were separately used to compare the statistical differences. The Kaplan-Meier method was used to estimate the overall survival (OS) of HCC patients with high or low level of ZNF521. Difference was considered statistically significant when *P* < 0.05.

## Results

### ZNF521 is down-regulated in HCC tissues and cells

**Figure 1:**
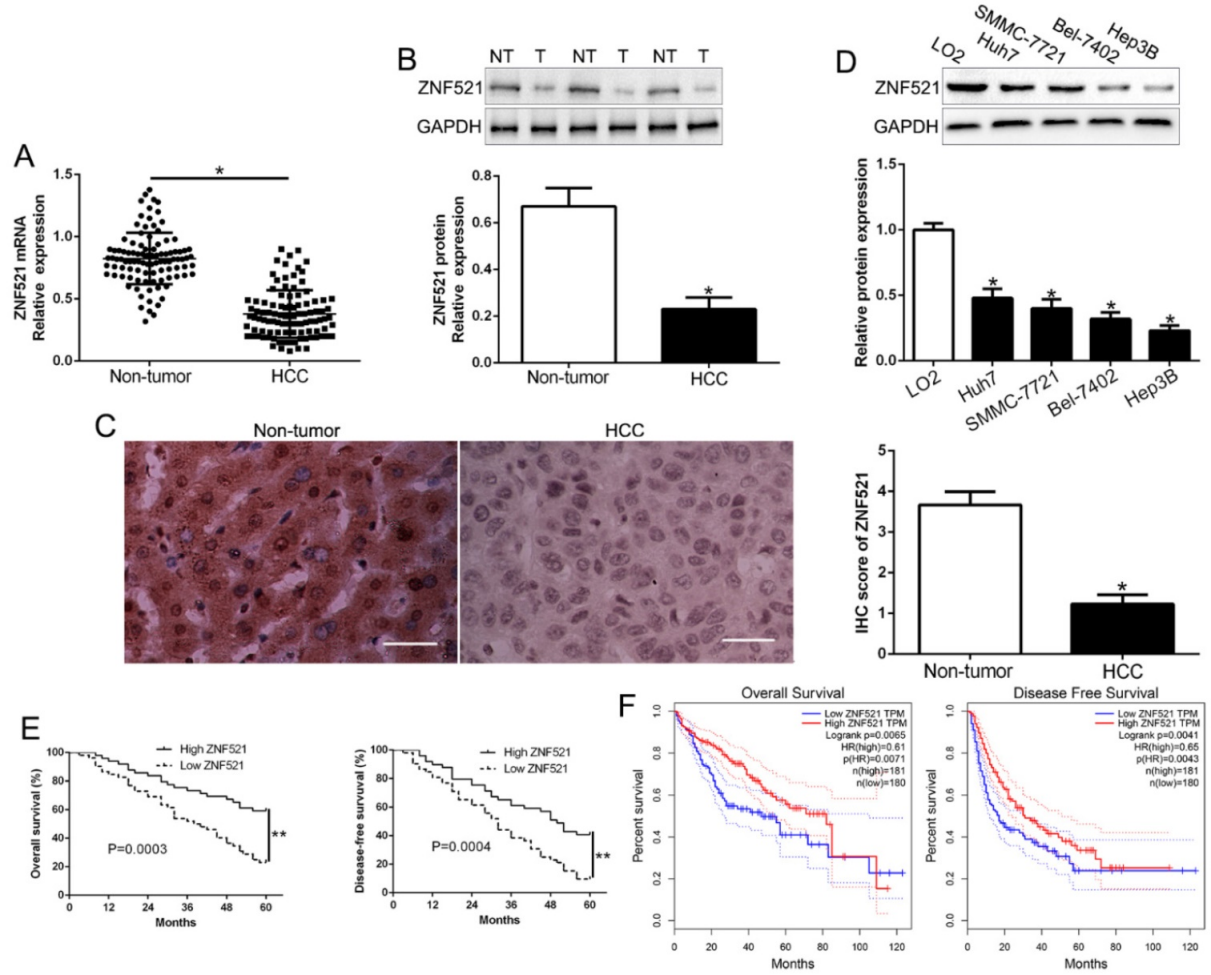
** ZNF521 is significantly down-regulated in HCC tissues and cell lines.** (A) Relative ZNF521 mRNA expression levels in HCC tissues and matched adjacent nontumor tissues were determined by qRT-PCR. (B) Representative Western blot analysis of ZNF521 expression in the HCC (T) and nontumor tissues (NT) was shown. (C) Representative images of IHC staining of ZNF521 in HCC and adjacent non-tumor tissues. Comparing differences in the expression level of ZNF521 protein (D) between HCC cell lines with the immortalized normal hepatic cell LO2. (E) HCC patients with lower expression of ZNF521 had worse overall survival and disease-free survival. (F) Cases with high/low ZNF521 expression were analyzed. n = three repeats with similar results; *P < 0.05.

To determine the function of ZNF521 in HCC progression, the expression of ZNF521 mRNA and protein was determined by qRT-PCR and western blot. The results revealed that ZNF521 mRNA and protein was robustly reduced in HCC tissues relative to that in adjacent non-tumor tissues (*P* < 0.05, Figure 1A-B). Moreover, IHC staining assays showed that ZNF521 IHC scores in HCC tissues was down-regulated compared to normal tissues (*P* < 0.05, Figure 1C). In HCC cells, our results showed that ZNF521 was decreased in HCC cells compared with normal hepatic cells LO2 (*P* < 0.05, Figure 1D). Therefore, we demonstrated that ZNF521 plays a role as a tumor suppressor in HCC progression.

### ZNF521 expression is associated with malignant clinic-pathological characteristics

To clarify the role of ZNF521 in clinical, we divided the HCC patients as different subgroups according to the median value as a cutoff. The down-regulated ZNF521 was significantly associated with large tumor size (*P* = 0.026) and advanced TNM stage (*P* = 0.018, Table 1). Kaplan-Meier survival analysis cure showed that low ZNF521 HCC patients had a shorter overall survival (OS) and disease-free survival (DFS) in HCC patients (*P* = 0.0003, 0.0004, respectively, Figure 1E). Moreover, in other cohort HCC patients, 182 patients with ZNF521 high expression and 182 patients with ZNF521 low expression were analyzed in GEPIA (*P* < 0.05, Figure 1F). These data suggest that ZNF521 is a potential prognostic biomarker in HCC patients.

### ZNF521 inhibits cell proliferation, colony formation and promotes apoptosis in HCC

**Figure 2:**
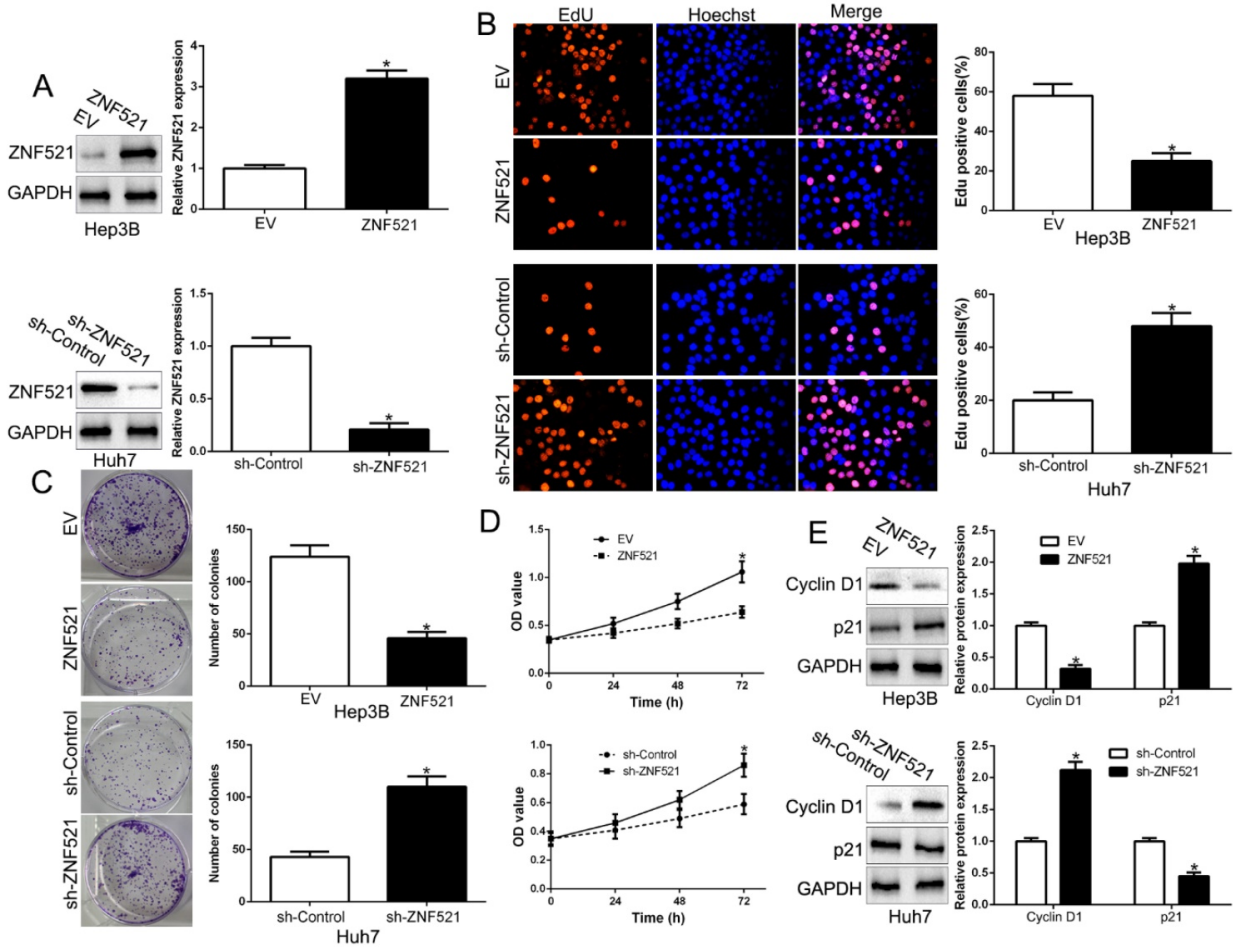
** ZNF521 inhibited cell proliferation, colony formation and viability *in vitro.*** (A) Hep3B and Huh7 cells that were transfected with different vectors were subjected to western blot for ZNF521 expression. ZNF521 overexpression inhibited cell proliferation (B), colony formation (C) and viability (D) in Hep3B cells, while ZNF521 knockdown promoted proliferation (B), cell colony formation (C) and viability (D) in Huh7 cells. (E) Western blot analysis of protein Cyclin D1 and p21 expression in the presence and absence of ZNF521. *P<0.05. EV, empty vectors. n=three independent experiments; *P<0.05.

To clarify the function of ZNF521 in HCC, we performed gain- and loss-of function assays by transfecting respective vectors in Hep3B and Huh7 whose endogenous ZNF521 was lowest and highest expression in HCC cell lines (*P* < 0.05, Figure 2A). We used Edu, colony formation and CCK-8 assay showed that ZNF521 overexpression significantly inhibited cell proliferation, colony formation and cell viability in Hep3B cells (*P* < 0.05, Figure 2B-D). Conversely, ZNF521 knockdown showed opposite effect on Huh7 cells (*P* < 0.05, Figure 2B-D). In addition, western blot revealed that ZNF521 regulated Cyclin D1 and p21 expression (*P* < 0.05, Figure 2E). These data proposed that ZNF521 hindered cell growth in HCC cells *in vitro*.

### ZNF521 inhibits HCC growth *in vivo*

**Figure 3:**
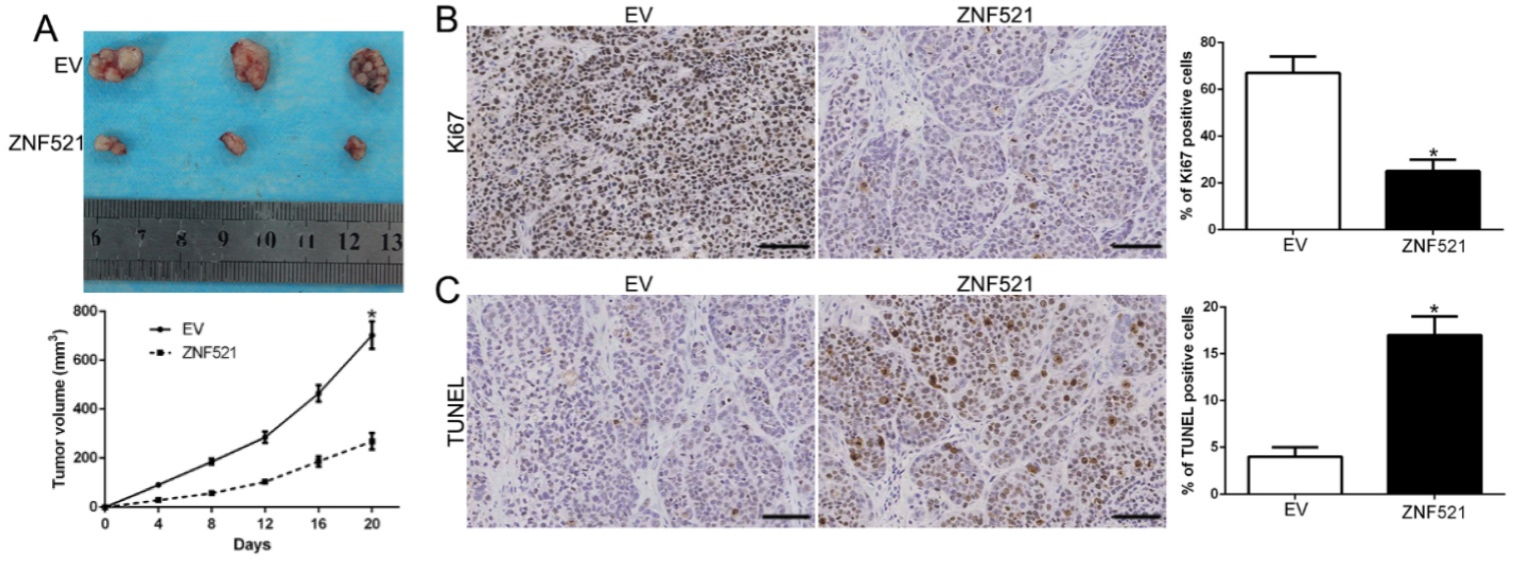
** ZNF521 inhibited tumor growth *in vivo.*** (A) Tumor growth curve revealed that ZNF521 overexpression significantly inhibited tumor growth *in vivo*. (B) Tumor nodules were subjected to immunohistochemical staining for Ki-67 assays and (C) TUNEL and quantitative analysis. Scale bar: 70 µM; *P < 0.05.

To further explore the function of ZNF521 on tumor growth *in vivo*, we used the subcutaneous tumor model and the data showed that ZNF521 overexpression significantly inhibited the tumor growth than control cells in mice (*P* < 0.05, Figure 3A). Moreover, we used the Ki67 and TUNEL staining to evaluate the proliferative and apoptotic rate *in vivo*. ZNF521 overexpression decreased Ki67 positive staining rate and increased the number of apoptotic cells for TUNEL positive staining (*P* < 0.05, Figure 3B-C). These results indicated that ZNF521 suppressed tumor growth *in vivo*.

### ZNF521 antagonizes Runx2 transcriptional activity in HCC cells

**Figure 4:**
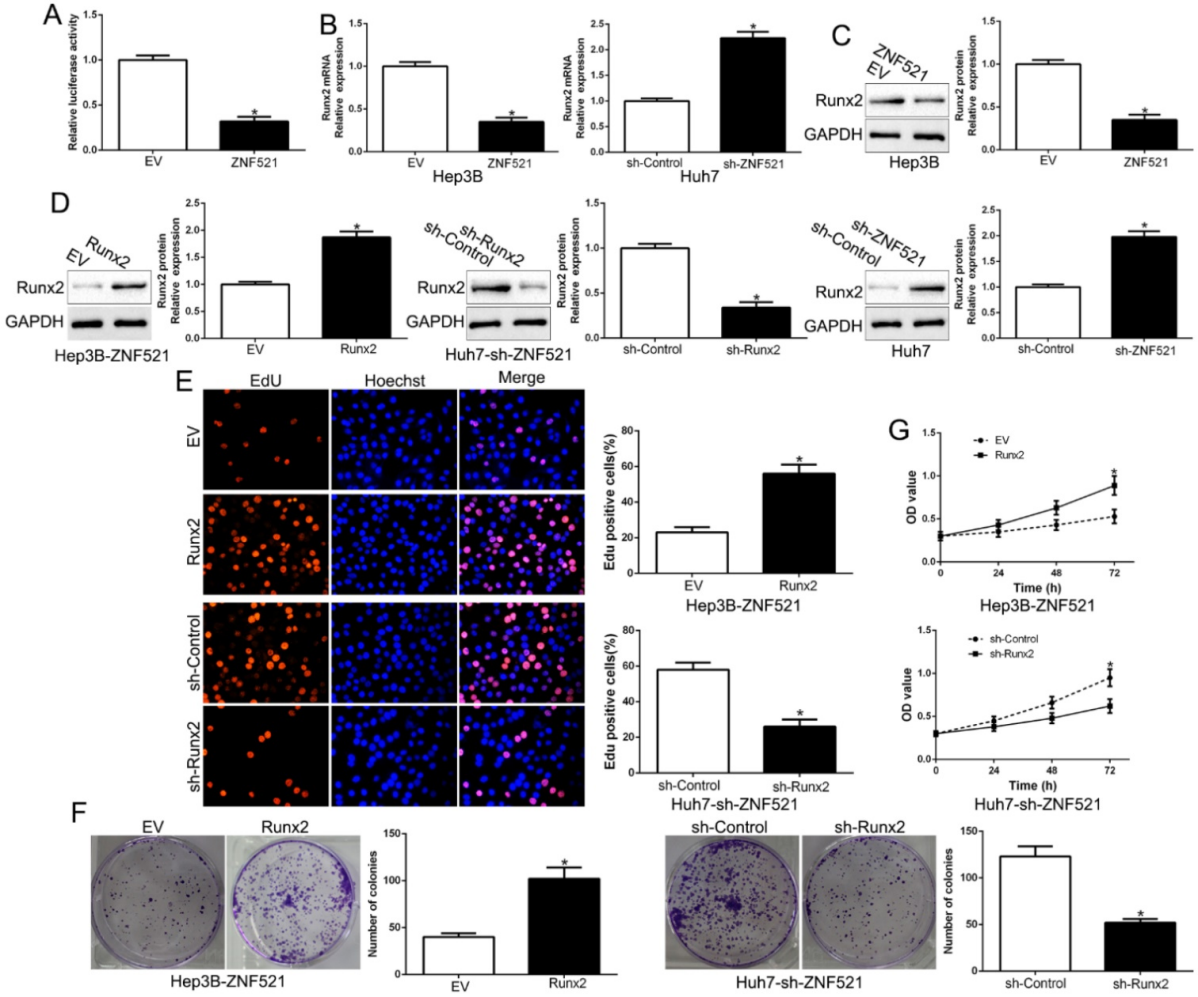
** ZNF521 antagonizes Runx2 transcriptional activity in HCC cells.** (A) ZNF521 inhibited Runx2 transcriptional activity. ZNF521 overexpression inhibited Runx2 mRNA (B) and protein (C) expression while ZNF521 knockdown promoted Runx2 mRNA (B) and protein (C) expression. (D) ZNF521-overexpressing Hep3B cells that were transfected with Runx2 overexpression vectors and ZNF521-supressing Huh7 cells that were transfected with Runx2 knockdown vectors were subjected to immunoblotting for Runx2. Runx2 overexpression reversed the inhibitory effects on cell proliferation (E), colony formation (F) and viability (G) of ZNF521-overexpressing Hep3B cells; *P < 0.05.

Runt-related transcription factor 2 (Runx2) is a key promoter of HCC 17. We examined the effects of ZNF521 on Runx2-induced of Runx2-luciferase reporter genes in Hep3B. As shown in Figure 4A, ZNF521 strongly repressed Runx2 transcriptional activity (*P* < 0.05, Figure 4A). Moreover, ZNF521 overexpression inhibited Runx2 mRNA and protein expression while ZNF521 knockdown increased Runx2 mRNA and protein expression (*P* < 0.05, Figure 4B-C). To confirm that Runx2 mediated the effects on ZNF521 on HCC cells, we reversed Runx2 expression in HCC cells (*P* < 0.05, Figure 4D). Runx2 reversed the effects of ZNF521 on HCC cells (*P* < 0.05, Figure 4E-G). Runx2 was up-regulated in HCC compared to adjacent non-tumor tissues (*P* < 0.05, Figure S1). These results revealed that ZNF521 repressed Runx2 transcriptional activity and exerted its effects at least partly through Runx2.

### AKT phosphorylation acts downstream of ZNF521 to mediate its effects on HCC

**Figure 5:**
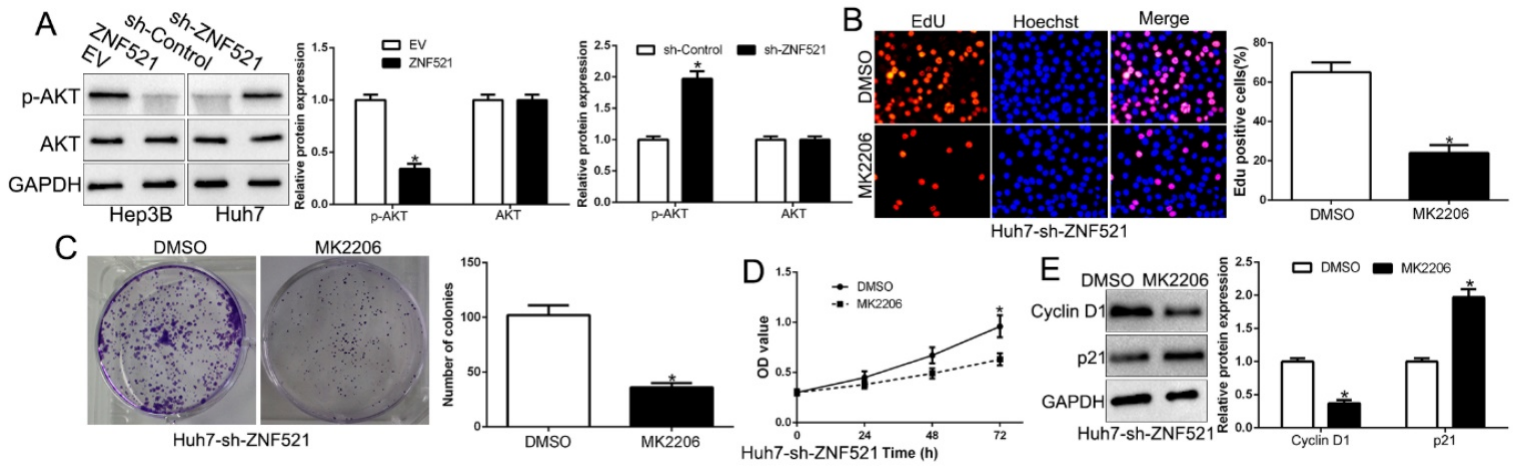
** AKT phosphorylation acts downstream of ZNF521 to mediate the effects on HCC cells.** (A) ZNF521 overexpression inhibited AKT phosphorylation while ZNF521 knockdown promoted AKT phosphorylation. AKT phosphorylation inhibitor MK2206 reversed the promotive effects on cell proliferation (B), colony formation (C), viability (D) and related-factors expression (E) of ZNF521-suppressing Huh7 cells; *P < 0.05.

AKT phosphorylation signaling was involved in HCC development 18. We demonstrated that ZNF521 overexpression inhibited AKT phosphorylation while ZNF521 knockdown increased the AKT phosphorylation (*P* < 0.05, Figure 5A). Moreover, we inquired whether AKT phosphorylation is necessary for ZNF521-induced effects on HCC by using the AKT inhibitor MK2206. We found that ZNF521 knockdown induced cell proliferation, colony formation and cell viability of HCC cells was significantly reversed by the addition of MK2206 (*P* < 0.05, Figure 5B-E). These data suggest that AKT signaling is necessary for ZNF521-induced effects of HCC cells.

### ZNF521 expression is regulated by miR-802

**Figure 6:**
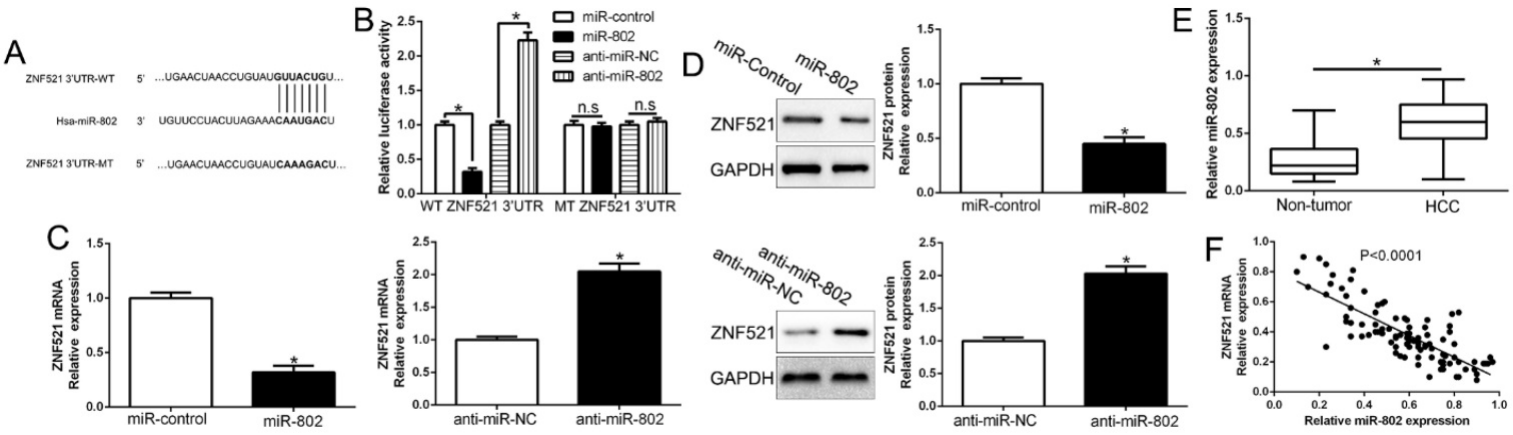
** ZNF521 is identified as a direct target of miR-802 in HCC.** (A) miR-802 and its putative binding sequence in the 3'-UTR of ZNF521. (B) miR-802 significantly suppresses the luciferase activity that carried wild-type (wt) but not mutant (mt) 3'-UTR of ZNF521. Anti-miR-802 led to a notable increase in the luciferase activity of wt 3'-UTR of ZNF521. (C) qRT-PCR analysis of ZNF521 mRNA expression in Huh7 cells with miR-802 or miR-control vector transfection and Hep3B cells with anti-miR-802 or anti-miR-NC vector transfection. (D) Overexpression of miR-802 reduced the expression of ZNF521 protein in Huh7 cells and knockdown of miR-802 increases the level of ZNF521 protein in Hep3B cells. (E) The expression level of miR-802 in HCC tissues and adjacent non-tumor tissues. (F) A significant inverse correlation between the mRNA levels of ZNF521 and miR-802 was observed in HCC tissues. U6 as the internal control; *P < 0.05.

Growing evidences indicated that microRNAs play crucial roles in cancer development and progression by targeting mRNAs for degradation or translational repression 19. To investigate the upstream that ZNF521 was down-regulated, we searched the database miRNA.org, TargetScan and PicTar to predict that miR-802 bind to the 3'UTR of ZNF521 (Figure 6A). We used luciferase reporter assays to show that miR-802 overexpression markedly decreased while miR-802 knockdown increased the luciferase activity of HCC cells with wild-type ZNF521 3'UTR (*P* < 0.05, Figure 6B). However, the activity in mutant-type ZNF521 3'UTR had no change (Figure 6B). Moreover, miR-802 overexpression significantly inhibited while miR-802 knockdown promoted ZNF521 mRNA and protein in HCC cells (*P* < 0.05, Figure 6C-D). In HCC tissues, our results showed that miR-802 was up-regulated in HCC tissues compared to adjacent non-tumor tissues (*P* < 0.05, Figure 6E), which was consistent with previous studies 20. We also confirmed an inverse correlation between ZNF521 mRNA and miR-802 in HCC tissues (*r*=-0.7223, *P* < 0.05, Figure 6F). Moreover, miR-802 overexpression significantly promoted cell proliferation, colony formation and cell viability in Huh7 cells (*P* < 0.05, Figure S2A-D). Conversely, miR-802 knockdown showed opposite effects in Hep3B cells (*P* < 0.05, Figure S2A-D). In conclusion, we demonstrated that miR-802 regulated ZNF521 expression in HCC tissues.

## Discussion

Zinc finger structures, forming a total of 6 zinc finger clusters, participate in multiple signaling pathways and regulate the downstream target genes, thus regulating various biological processes such as hematopoietic differentiation, cell proliferation and autophagy 21. ZNF521 was characterized as potent inhibitor of EBF1 and contributed to the development of B-cell leukemias. ZNF521 was a target gene and key effector of parathyroid hormone-related peptide signaling in growth plate chondrocytes 22. In this research, we demonstrated that ZNF521 was down-regulated in HCC tissues and cells for the first time. In clinical data, we showed that down-regulated ZNF521 was significantly associated with large tumor size and advanced TNM stage. Moreover, ZNF521 down-regulation showed a worse prognosis for 5-year OS and DFS in HCC patients, which was consistent in other cohort HCC patients. These findings confirmed the fundamental role of ZNF521 in HCC development.

To investigate the biological effects of ZNF521 in HCC, we used the gain- and loss-of function experiment to clarify that ZNF521 significantly inhibited cell proliferation, colony formation and cell viability in HCC cells *in vitro* and *in vivo*. Mechanically, we confirmed that ZNF521 exerted its effects by regulating Runx2 expression and repressed its transcriptional activity. Runx2 was involved in osteoblastic differentiation, skeletal morphogenesis and cancer progression 23-25. Runx2 overexpression promoted CXCR7 expression and cellular trafficking, AKT hyperactivation and prostate tumorigenesis 26. Runx2 plays an oncogenic role in esophageal carcinoma by activating the PI3K/AKT and ERK signaling pathways 27. Runx2 promoted HCC cell migration and invasion by regulating MMP9 expression 17. Here, we identified that Runx2 mediated the function of ZNF521 on HCC cells. Moreover, PI3K/AKT signaling was proposed as a vital mechanism that regulates the initial steps of metastatic progression of cancer 12, 28. Our results showed that AKT pathway abolished the inhibitory effect of ZNF521 on HCC cells. Taken together, these data demonstrated the suppressive effect of ZNF521 was mediated by inhibiting AKT phosphorylation pathway in HCC.

Previous studies showed that miRNA can specifically induce the degradation of target gene mRNA and inhibit the translation of gene 29, 30. Here, we confirmed that miR-802 could bind with ZNF521 3'UTR. miR-802 negatively regulated the expression of ZNF521 mRNA and protein in HCC cells. Previous studies reported that miR-802 accelerates hepatocellular carcinoma growth by targeting Runx3 20. High blood miR-802 is associated with poor prognosis in HCC patients by regulating DNA damage response 1 (REDD1)-mediated function of T cells 31. miR-802 inhibits cell proliferation and induces apoptosis in human laryngeal cancer by targeting Camp-regulated phosphoprotein 19 32. These data further confirm that miR-802 exerted its effects was cell-specific 33, 34. Here, we demonstrated that miR-802 promoted HCC growth. In conclusion, these data suggest that ZNF521 was a downstream target of miR-802 in HCC.

In summary, we reported for the first time that ZNF521 was down-regulated in HCC tissues and cells and its down-regulation was associated with malignant clinical features and unfavorable prognosis. We confirmed that ZNF521 inhibited cell proliferation, colony formation and cell viability through Runx2 transcriptional inhibition and AKT phosphorylation pathway. Moreover, miR-802 regulated ZNF521 expression in HCC cells. These data indicated that ZNF521 was an important biomarker of HCC progression and a novel and attractive therapeutic target for HCC treatment.

## Supplementary Material

Supplementary figures and tables.

## Figures and Tables

**Table 1:** Clinical correlation of ZNF521 expression in HCC (n = 101)

Clinical parameters	Cases (n)	Expression level		*P* value (* *p*<0.05)
		ZNF521^high^ (n=49)	ZNF521^low^ (n=52)	
**Age (years)**				0.150
< 60 years	65	35	30	
≥60 years	36	14	22	
**Gender**				0.56
Male	80	40	40	
Female	21	9	12	
**Tumor size (cm)**				0.026*
< 5 cm	72	40	32	
≥ 5cm	29	9	20	
**Tumor number**				0.507
solitary	84	42	42	
multiple	17	7	10	
**Edmondson**				0.382
I+II	23	13	10	
III+IV	78	36	42	
**TNM stage**				0.018*
I+II	76	42	34	
III+IV	25	7	18	
**Vascular infiltration**				0.678
Present	16	7	9	
Absent	85	42	43	
**AFP**				0.216
< 400 ng/ml	24	9	15	
≥ 400 ng/ml	77	40	37	
**HBsAg**				0.444
positive	91	43	48	
negative	10	6	4	

HCC, hepatocellular carcinoma; AFP, alpha-fetoprotein; TNM, tumor-node-metastasis. *Statistically significant.
