# Supplementary material for: ZNF521 which is downregulated by miR-802 suppresses malignant progression of Hepatocellular Carcinoma through regulating Runx2 expression
Source: J Cancer. 2020 Aug 1;11(19):5831–9. doi: 10.7150/jca.45190 (PMC7477442; doi:10.7150/jca.45190)
Supplement: Supplementary file 1 — Supplementary figures and tables. [file jcav11p5831s1.pdf]

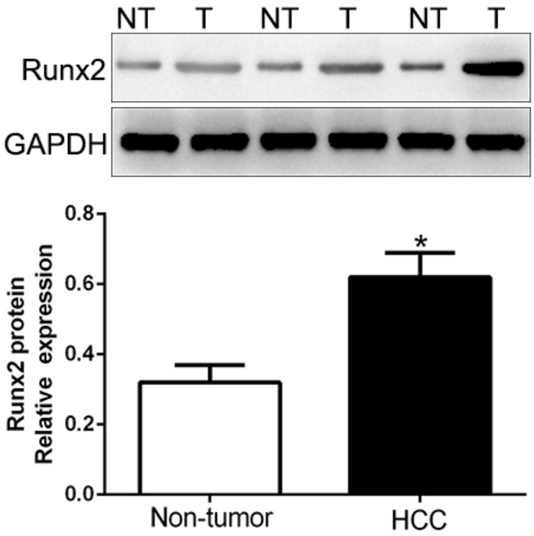

Sup.Fig.1. Runx2 is significantly up-regulated in HCC tissues. Representative Western blot analysis of Runx2 expression in the HCC (T) and nontumor tissues (NT) was shown.

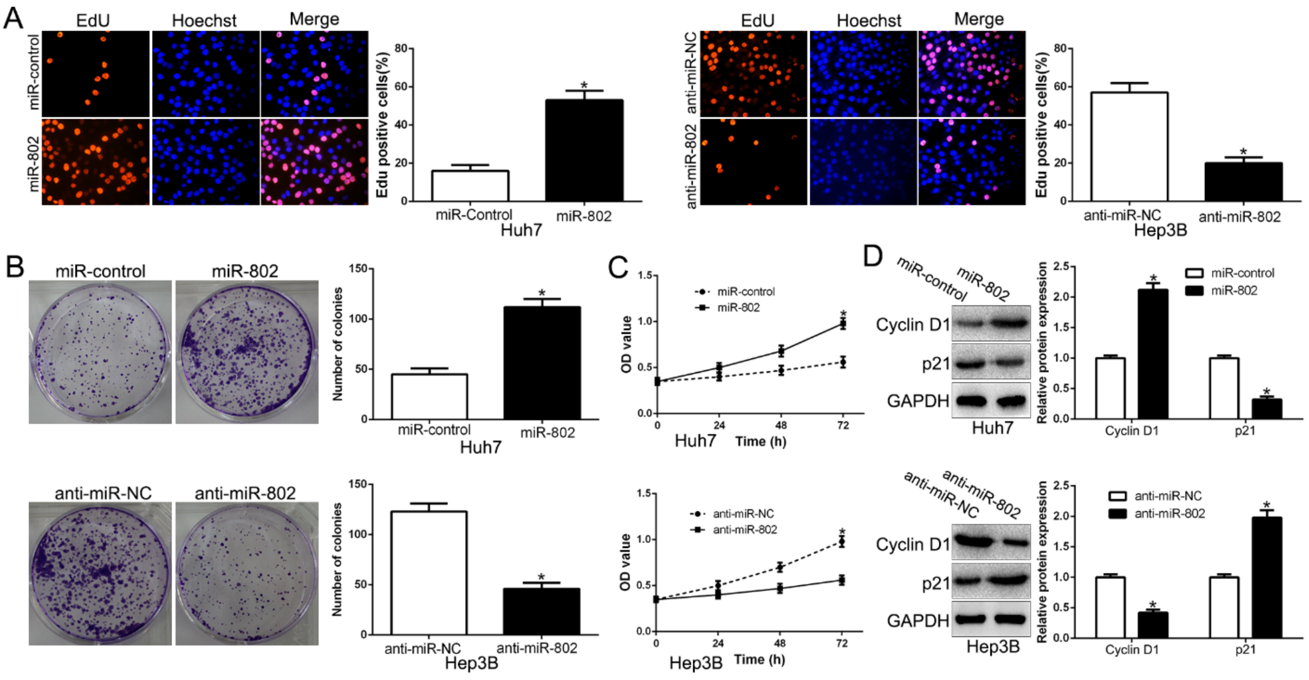

Sup.Fig.2 miR-802 promoted cell proliferation, colony formation and viability. miR-802 overexpression promoted cell proliferation (A), colony formation (B) and viability (C) in Huh7 cells, while miR-802 knockdown inhibited proliferation (A), cell colony formation (B) and viability (C) in Hep3B cells. (D) Western blot analysis of protein Cyclin D1 and p21 expression in the presence and absence of miR-802. *P<0.05. n=three independent experiments. *P<0.05.
